# Predictive Role of Metabolic Profiling in Rivaroxaban Efficacy for Thrombus Lysis in Atrial Fibrillation

**DOI:** 10.3390/ijms262110757

**Published:** 2025-11-05

**Authors:** Sylwia Michorowska, Natalia Korytowska-Przybylska, Roman Piotrowski, Piotr Kułakowski, Joanna Giebułtowicz

**Affiliations:** 1 Department of Drug Chemistry, Pharmaceutical and Biomedical Analysis, Faculty of Pharmacy, Medical University of Warsaw, 02-097 Warsaw, Poland; sylwia.solobodowska@wum.edu.pl (S.M.); natalia.korytowska@wum.edu.pl (N.K.-P.); 2 Department of Cardiology, Postgraduate Medical School, Grochowski Hospital, 04-073 Warsaw, Poland; rpiotrow@op.pl (R.P.); kulak@kkcmkp.pl (P.K.)

**Keywords:** atrial fibrillation, metabolomic profiling, acylcarnitines, rivaroxaban, thrombus

## Abstract

Traditional anticoagulants used in atrial fibrillation (AF) are being increasingly replaced by novel oral anticoagulants such as rivaroxaban, improving patient outcomes. Although rivaroxaban 20 mg/1× daily is approved to reduce stroke and systemic embolism risk in AF, some patients still develop thrombus in the left atrial appendage (LAA). A previous study demonstrated thrombus lysis with a modified regimen of rivaroxaban 15 mg/2× daily, yet over 50% of patients remained unresponsive despite therapeutic plasma levels. This study compared metabolic profiles of responders and non-responders to identify predictive markers of treatment efficacy. From the RIVA-TWICE study cohort (*n* = 249), 15 AF patients with LAA thrombus despite standard dosing were switched to 2 × 15 mg rivaroxaban. Plasma samples collected prior to dose modification underwent untargeted and targeted LC-MS analysis, focusing on acylcarnitines (ACs), carnitine, and its precursors. Thrombus resolution occurred in 7 (46.7%) patients, who showed differential abundance of metabolites related to alpha-linolenic acid and fatty acid metabolism, carnitine synthesis, and arginine/proline pathways. Targeted analysis confirmed elevated levels of ACs, carnitine, and precursors. Findings suggest that a patient phenotype, including carnitine, its precursors, and ACs, may predict rivaroxaban efficacy in thrombus lysis. While these metabolites may not directly mediate lysis, their elevated levels represent potential biomarkers of treatment response.

## 1. Introduction

Atrial fibrillation (AF) is an increasingly common cardiovascular arrhythmia prevalent across the globe. It is associated with an increased risk of atrial thrombi (most commonly in the left atrial (LA) and left atrial appendage (LAA)) [1], stroke, and death [2]. The 2019 global burden of AF estimated 59.7 million prevalent cases [3]. Numbers are the highest in countries with a high socio-demographic index. However, the largest recent increase has been noticed in the middle socio-demographic index countries [4]. Importantly, future estimations suggest an increase at a global level mainly due to aging of the worldwide population and population growth. Improved health care and thromboembolism prophylaxis is therefore a must [3]. Systematic use of anticoagulants has been shown to result in a decreased absolute number of ischemic strokes [2].

Vitamin K antagonists (e.g., warfarin), despite being effective and convenient (taken orally), have many disadvantages including unpredictable pharmacokinetics and pharmacodynamics, a slow onset of action, a narrow therapeutic window, multiple food–drug and drug–drug interactions as well as inter- and intraindividual variability in dose response [5]. Non-vitamin K antagonist oral anticoagulants (NOACs), directly inhibiting either thrombin (dabigatran) or factor Xa (rivaroxaban, apixaban) of the coagulation cascade, were shown to be superior to warfarin in the prevention and treatment of thrombosis [1]. Rivaroxaban, a NOACs direct, reversible and dose-dependent inhibitor of free and bound factor Xa, was shown to be safe and, most importantly, more effective than warfarin at resolving LA/LAA thrombus in nonvalvular AF patients taking 20 mg of the drug once daily [1]. The selection of such a dose regimen was a result of the dose-finding studies which revealed similar efficacy and safety profiles of once-daily and twice-daily regimens [6]. Based on the collected results once-daily rivaroxaban 20 mg was chosen to be assessed in nonvalvular atrial fibrillation in ROCKET-AF (Rivaroxaban-once daily, oral direct factor Xa inhibition compared with vitamin K antagonism for prevention of stroke and embolism trial in atrial fibrillation) study [7] and approved for the reduction in the risk of stroke and systematic embolism in patients suffering from nonvalvular atrial fibrillation by the European Medicines Agency in September 2011 [8] and by the Food and Drug Administration (FDA) in November 2011 [9]. Moreover, the European Society of Cardiology (ESC) recommends NOACs in preference to vitamin K antagonist for AF patients [10]. However, in some patients, standard rivaroxaban treatment was shown to be ineffective, despite excellent compliance (20 mg once daily for 8 weeks) [11], posing a risk of thromboembolic events and therefore indicating an urgent need for alternative therapeutic approaches, e.g., modified dose regimen. The RIVA-TWICE study [12], which monitored 249 patients, demonstrated complete resolution of thrombus in the LAA in some patients being given rivaroxaban 15 mg twice daily for 8 weeks, who did not respond to the earlier, standard 20 mg once a day therapy. The treatment with an increased dose (15 mg twice daily) was, however, not fully successful, as 53.3% of patients did not respond to it. The only statistically significant differences between the patients in whom thrombus persisted despite twice-daily therapy and those in whom it was resolved were CHA_2_DS_2_-VASc (4.0 ± 1.5 vs. 2.3 ± 1.5, respectively) and HAS-BLED scores (1.8 ± 0.7 vs. 0.7 ± 0.8, respectively), as well as mean LAA emptying velocity (0.18 ± 0.02 m/s and 0.27 ± 0.09 m/s, respectively). Importantly, no statistically significant difference in anti-Xa activity (used to monitor anticoagulant therapy) between the two study groups was observed. Moreover, all patients had therapeutic rivaroxaban plasma concentrations as assessed using the anti-factor Xa chromogenic assay calibrated for rivaroxaban therapeutic window [12], meaning that there were some other factors affecting the success of the therapy with increased dose. Understanding the underlying causes is therefore important to ensure effective and best patient-matched therapy. That is why the aim of this study was to compare the metabolic profiles of 15 patients who were selected from the 249 participants in the RIVA-TWICE study [12], including responders and non-responders to a modified rivaroxaban dosage regimen and identify differentially abundant metabolites which may account for the observed differences in the effectiveness of the therapy.

## 2. Results and Discussion

### 2.1. Concentration of Rivaroxaban in Plasma

All the variants of QuEChERS method gave similar results of recovery (91–97%) and matrix factor (93–100%), so the cheapest variant was selected. The calibration curve obtained by weighted linear regression analysis was linear in the range 1.95–1000 ng/mL regarding the peak area ratio of rivaroxaban and the internal standard versus the nominal concentration of rivaroxaban (R^2^ = 0.997). The accuracy for the lower limit of quantitation (LLOQ, 1.95 ng/mL) was 109% (RSD = 4.5%, *n* = 12) within three runs, and 106–114% (RSD = 2.1–5.6%, *n* = 3) within one day. The accuracy for the QC_low_ (5 ng/mL) samples between runs was 111% (RSD = 4.7%, *n* = 6) and within one day was 110% (RSD = 0.8%, *n* = 3). The inter-day accuracy for QC_medium_ (500 ng/mL) was 111% (RSD 5.1%, *n* = 9), whereas for QC_high_ (750 ng/mL) it was 114% (RSD = 2.4%, *n* = 9). The intra-day accuracy for QC_medium_ was 105% (RSD = 4.3%, *n* = 3), whereas for QC_high_ it was 110% (RSD = 0.6%, *n* = 3). The matrix factors for different lots of plasma for rivaroxaban and the internal standard were 98- 117% and 97–107% for the QC_low_, and 96–102% and 97–100% for QC_high_, respectively. The CV of the IS-normalized matrix factor was 4.1% for QC_low_ and 1.5% for QC_high_, respectively.

**Figure 1:**
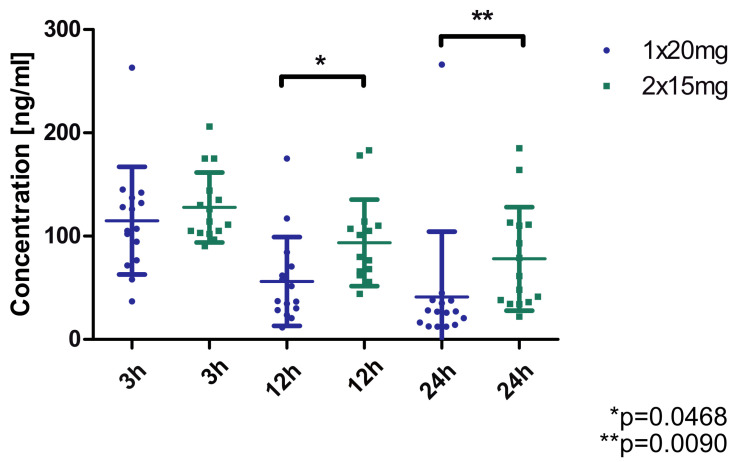
Concentration of rivaroxaban in plasma of patients (*n* = 15) treated with standard (1 × 20 mg) and modified dose (2 × 15 mg). * *p* = 0.0468, ** *p* = 0.0090 (Wilcoxon matched-pairs test).

The plasma concentration of rivaroxaban was measured in patients with nonvalvular atrial fibrillation (15 patients selected from 249 participants of the RIVA-TWICE study [12]) before and after starting the 15 mg twice-daily dosing regimen, at 3, 12 and 24 h. The results are shown in Figure 1 and indicate a consistently higher concentration at each time-point for the 2 × 15 mg dosing regimen compared to the 1 × 20 mg regimen. Statistically significant differences were obtained between the two regimens at 12 and 24 h.

**Figure 2:**
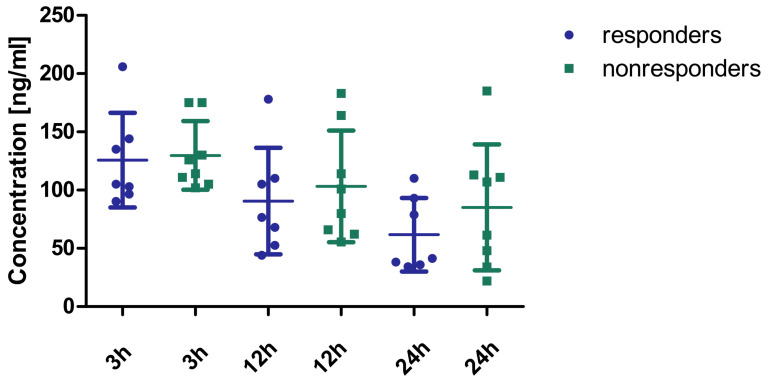
Concentration of rivaroxaban in the plasma of patients effectively (*n* = 7) and non-effectively (*n* = 8) treated with a modified dose (2 × 15 mg). No statistically significant differences were observed between the two groups (3 h: Mann–Whitney U test; 12 h, 24 h: Student’s *t*-test). The concentrations tended to be higher in responders compared to non-responders, especially at 12 h and 24 h post-administration. However, it is important to emphasize that all concentrations were within the recommended concentration ranges set by the International Council for Standardization in Hematology [13] and the European Heart Rhythm Association [14].

The concentrations of rivaroxaban in the plasma of patients who responded (*n* = 7) and who did not respond (*n* = 8) to rivaroxaban 2 × 15 mg are shown in Figure 2. No statistically significant differences were observed between effectively treated and non-effectively treated patients. Due to the absence of observable differences in drug concentrations between responders and non-responders, the decision was made to identify phenotypic distinctions between the groups. This step was crucial for predicting whether adjusting the dosage for a specific patient is likely to yield a positive clinical outcome. To achieve this objective, the method of metabolic fingerprinting was employed to analyze plasma samples collected 24 h prior to the dosage modification.

### 2.2. Untargeted Metabolomics

**Figure 3:**
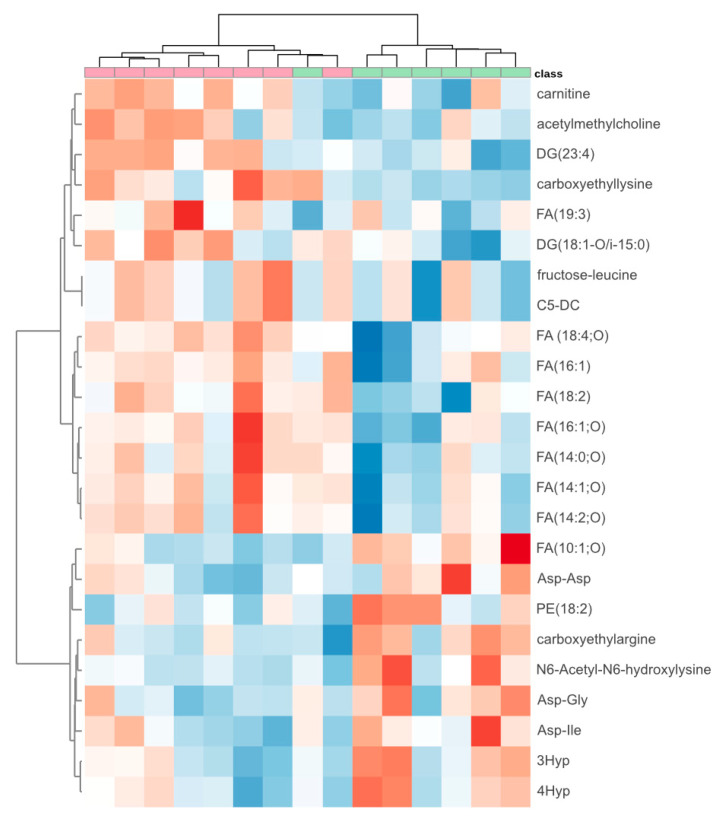
Heatmap of 24 compounds that differed significantly between patients effectively (*n* = 7) (green) and non-effectively (*n* = 8) (red) treated with a modified dose of rivaroxaban. The deeper the blue color, the lower the level of the metabolite. The deeper the red color, the higher the level. The metabolites with similar abundance patterns across the samples were clustered together, and patients with similar metabolic fingerprints were also grouped into clusters. DG: diglyceride; C5-DC: glutarylcarnitine; FA: fatty acid; PE: phosphatidylethanolamine; 3Hyp: 3-hydroxyproline; 4Hyp: 4hydroxyproline.

We detected 62 compounds on the HILIC column and 42 compounds on the PFP column that exhibited significant differences between patients responding effectively and those not responding effectively to the modified dose of rivaroxaban. However, we were able to tentatively propose structures for only 24 of them, primarily due to the absence or low quality of fragmentation spectra for most detected compounds. The heatmap depicting these compounds is presented in Figure 3. The Quantitative Enrichment Analysis identified several significantly enriched pathways (FDR < 0.05): alpha linolenic acid and linoleic acid metabolism, oxidation of branched chain fatty acids, fatty acid metabolism, β-oxidation of very long-chain fatty acids, carnitine (CAR) synthesis, mitochondrial β-oxidation of short-chain saturated fatty acids, mitochondrial β-oxidation of long-chain saturated fatty acids, fatty acid biosynthesis and arginine and proline metabolism. We concluded that the ineffective treatment with the modified rivaroxaban dose may be related to altered fatty acid metabolism. Imbalanced fatty acid metabolism occurs, for example, in heart failure, due to a decrease in fatty acid oxidation (FAO), which is the primary source of energy production for a healthy heart [15]. An important indicator of FAO disorders, which mainly affect high energy-requiring organs including the heart, is the plasma level of acylcarnitines (ACs) [16].

ACs are a group of metabolites that, among other functions, supply fatty acids for β-oxidation in the mitochondrial matrix. While short-chain ACs are generated from glucose, amino acids, and the degradation of fatty acids, medium- (MCACs) and long-chain ACs (LCACs) are solely derived from fatty acid metabolism [17]. Under physiological conditions, 60–90% of the energy required for myocardial function comes from fatty acid oxidation [18]. The myocardium has limited reserves of fatty acids, so to meet its energy needs, they are constantly taken up from the blood and transported into myocardial mitochondria as ACs. It has been shown in laboratory animals that the level of ACs in the heart correlates with their level in the blood [17]. Therefore, blood is an appropriate material for studying disorders of energy metabolism in cardiomyocytes.

ACs are generated within mitochondria or peroxisomes through the enzymatic action of carnitine acyltransferases on carnitine and acyl-CoAs [17]. Carnitine not only transports fatty acids to the mitochondria but also participates in removing fatty acids of various chain lengths from the mitochondria, which can be toxic in excess. Elevated levels of plasma ACs may indicate impaired β-oxidation of fatty acids and are associated with an increased frequency of cardiovascular diseases, such as coronary artery disease and heart failure [19]. The ACs profile also allows for the prediction of complications and mortality in heart failure and the occurrence of ischemic stroke. An increased level of ACs is an adverse prognostic factor in both pathologies [20,21]. This phenomenon is attributed to the association of ACs with oxidative stress, mitochondrial changes, and/or inflammation [22,23]. Dysregulations in the metabolism of fatty acids affect the activation of blood platelets, increase oxidative stress, and stimulate the synthesis of fibrinogen [21]. Therefore, for targeted quantitative analysis, short- (acetylcarnitine (C2), propionylcarnitine (C3), butyrylcarnitine (C4)), medium- (octanoylcarnitine (C8), dodecanoylcarnitine (C12)), long- (myristoylcarnitine (C14), palmitoylcarnitine (C16), octadecanoylcarnitine (C18)), branched- (isovalerylcarnitine (iC5)), unsaturated- (oleoyl-L-carnitine (C18:1), linoleoyl carnitine (C18:2)) and hydroxyl-/dicarboxyl-chain acylcarnitines (3-hydroksybutyrylcarnitine (C4-OH), glutarylcarnitine (C5-DC), hydroxyisovalerylcarnitine (iC5-OH), methylglutarylcarnitine (C6-DC), hydroxypalmitoylcarnitine (C16-OH)) [24] were chosen. Additionally, carnitine and its four precursors—methionine, trimethyllysine, butyrobetaine and lysine—were also determined.

### 2.3. Targeted Metabolomics

The primary source of carnitine is dietary absorption, although its synthesis can also occur through biosynthetic pathways in the human liver, kidney, and brain. Lysine, serving as the foundational carbon structure for carnitine, undergoes methylation, with the three methyl groups deriving from methionine to yield trimethyllysine. Subsequently, four enzymes play a crucial role in facilitating this biochemical transformation, with the final step being the hydroxylation of butyrobetaine into carnitine, catalyzed by γ-butyrobetaine dioxygenase (BBD) [25,26]. The synthesis of ACs within the organism typically involves the conjugation of a fatty acid with L-carnitine facilitated by the carnitine acyltransferase system. Carnitine acyltransferases, comprising distinct enzymes, contribute to the transfer of acyl groups characterized by varying chain lengths from acyl-CoA onto carnitine, thereby leading to the formation of short-, medium-, and long-chain ACs [24]. Plasma concentrations of carnitine, its precursors and selected ACs in patients successfully and unsuccessfully treated with modified dose of rivaroxaban are presented in Table 1.

**Figure 4:**
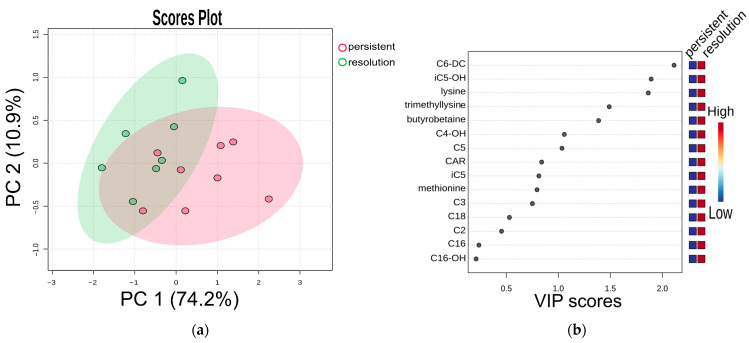
(**a**) Partial least squares-discriminant analysis (PLS-DA) and (**b**) VIP scores. Cross-validated PLS-DA score plot comparing metabolite profiles of 7 patients with effective treatment (group “resolution”) and 8 patients with ineffective treatment (group “persistent”), both receiving a modified rivaroxaban dose, shows the separation based on treatment results. The *p*-value based on permutation testing is 0.04. Light red and green ellipses represent 95% confidence intervals. Colored dots represent individual samples. The percentages given along the x and y axes are the scores of components 1 and 2, respectively.

High concentrations of methionine, trimethyllysine, butyrobetaine, lysine, C3, carnitine, iC5, iC5-OH, C6-DC, C18 correlated with thromboslysis. Partial least squares-discriminant analysis (PLS-DA) was used to assess the potential of the obtained metabolomic dataset for predicting treatment efficacy. We observed separation of groups in which rivaroxaban treatment was effective and ineffective (R2 = 0.43, Q2 = 0.25, permutation test *p* = 0.04). The compounds that affect separation the most are C6-DC, iC5-OH, lysine, trimethyllysine and butyrobetaine (Figure 4).

As mentioned earlier, we observed that patients in whom the thrombus was resolved had higher concentrations of carnitine and ACs, including short- (C3), long- (C18), branched- (iC5) chain, hydroxyl- (iC5-OH) and dicarboxyl- (C6-DC) ACs, as well as amino acids associated with carnitine biosynthesis, i.e., methionine, trimethyllysine, butyrobetaine and lysine. The elevated levels of these compounds may be related to the patient’s condition and ongoing disease.

Studies indicate that high level of LCACs may induce proarrhythmic effects and in consequence contribute to arterial thrombus formation due to altered cardiac blood circulation. Tests performed on HEK293 cells expressing hERG potassium channels, using LCACs (C18:1 and C16) at a concentration of approximately 1000 ng/mL, have shown that LCACs affect hERG potassium channels. These channels are vital for cardiomyocytes repolarization. Therefore, any disturbances in their activity can have a proarrhythmic effect [27]. Additionally, hERG potassium channels modulate excitation-contraction coupling and decrease conduction between cardiac cells [28]. However, the concentrations of LCACs detected in patients enrolled in our study were much lower, ranging from 8.6 (2.9) ng/mL (for C14) to 540 (252) ng/mL (for C18:2), compared to the concentration used in the above-mentioned study. Additionally, none of the LCACs mentioned above (C16 and C18:1) showed significant differences between our study groups.

The only SCAC whose level was significantly different in patients effectively and ineffectively treated with modified rivaroxaban dose was C3 acylcarnitine. This metabolite is mainly a breakdown product of two branched-chain amino acids, valine and isoleucine, after processing by branched-chain alpha-keto acid dehydrogenase [29]. Therefore, its higher level could indicate increased catabolism of these amino acids, possibly due to their elevated levels. In vitro studies have shown that valine and isoleucine, at concentrations much higher (1.17 mg/mL and 0.59 mg/mL, respectively) than physiological levels (circulating levels of valine at 0.02 mg/mL and isoleucine at 0.01 mg/mL) also have proarrhythmic effects attributed to the dysregulation of calcium homeostasis [30]. However, untargeted analysis did not reveal any significant differences in the level of branched-chain amino acids between the two study groups. This also suggests a low probability of distinct proarrhythmic activity due to either higher LCACs or branched-chain amino acid levels in one of our study groups.

Proarrhythmic effect has the potential to disrupt cardiac blood circulation, which is a critical factor in thrombus formation and resolution. It has been shown that flow velocities of 20 cm/s or less are linked to LAA thrombus formation and an increased risk of thromboembolic events. Moreover, resolution of thrombi in patients with AF treated with oral anticoagulants, among other drugs, was observed in those with significantly higher average LAA flow velocities during AF [31]. Patients treated effectively with a modified rivaroxaban dose exhibited a greater LAA emptying velocity (27 ± 9 cm/s) and a reduced count of procoagulant risk factors accounted for in the CHA_2_DS_2_-VASc and HAS-BLED scoring system [12], which may suggest better cardiac blood circulation compared to patients with persistent thrombus (18 ± 2 cm/s). This further supports the observation that the potential proarrhythmic effect related to the differences in ACs or branched-chain amino acids profile, which could lead to disrupted blood circulation, does not explain the lack of response of some patients to modified rivaroxaban treatment.

Furthermore, products of branched-chain amino acid catabolism, such as propionyl coenzyme A, have been shown to significantly promote platelet activity and arterial thrombus formation in mice via K255 tropomodulin-3 propionylation [32]. Given the significantly lower levels of propionyl acylcarnitine observed in patients who did not respond to modified rivaroxaban treatment, it is reasonable to suspect elevated levels of propionyl CoA and, consequently, greater platelet activity in these individuals. Rivaroxaban has been demonstrated to produce its antithrombotic effect through not only anticoagulant but also profibrinolytic mechanisms [33]. Additionally, it exerts an antiplatelet effect. In cardiovascular patients with nonvalvular AF receiving rivaroxaban the thrombin-induced platelet aggregation was reduced [34]. Therefore, patients with higher levels of propionyl CoA, may need a stronger anticoagulant and profibrinolytic activity, and thus even higher rivaroxaban doses.

The only LCAC exhibiting a significantly different level in effectively and non-effectively treated patients was C18. Some studies suggest that low plasma LCAC levels, which range from 3.09 to 437 ng/mL for different LCAC, are associated with the risk of venous thrombosis. It has been proven that LCACs possess anticoagulant properties (e.g., C18 at concentrations ranging from 1072 ng/mL to 2143 ng/mL) due to the inhibition of activated factor X [35]. Rivaroxaban also inhibits this factor [1], which suggests a potential synergistic effect. Despite the generally lower concentrations of C18 in patients enrolled in our study compared to the above-mentioned levels exerting anticoagulant activity, the level of C18 was significantly higher in patients with resolved thrombus, which potentially led to a greater anticoagulation effect and consequently thrombus lysis.

Elevated levels of ACs may not only be associated with the effectiveness of the treatment with modified rivaroxaban dose but also with the presence of various diseases. Specifically, C18 acylcarnitine, higher concentrations of which were detected in responders in our study, has been shown to significantly increase the diagnostic probability of non-ischemic dilated cardiomyopathy in heart failure patients [36]. Cardiomyopathy may lead to early mortality or impairment due to advancing heart failure, arrhythmia, stroke, or other embolic events. The link between cardiomyopathy and thromboembolism is due to various pathophysiological abnormalities, e.g., low cardiac output, dilated chambers, poor contractility, and endothelial dysfunction [37]. However, in our study, there were fewer cases of congestive heart failure among patients with resolved thrombus, despite higher levels of C18 acylcarnitine in this group of patients.

Patients in whom thrombus was resolved also had higher concentration of one of the dicarboxylacylcarnitines, C6-DC. In clinical studies, carnitines with two carboxyl groups (including C6-DC) have been associated with an increased risk of cardiovascular events [38,39]. In our study, five patients (two with resolved thrombus and three with persistent thrombus) had myocardial infractions or coronary artery disease. Therefore, cardiovascular events cannot explain the difference in C6-DC levels between responders and non-responders.

We have shown that carnitine precursors, such as trimethyllysine and butyrobetaine, are present in higher concentrations in patients in whom thrombus was resolved. High levels of these precursors have also been identified in patients with cardiovascular disease and are associated with negative prognostic implications. Additionally, these compounds might produce trimethylamine oxide (TMAO) [40], which may induce atrial fibrillation by affecting the cardiac autonomic nervous system, cell osmolarity, inflammation, and cardiac fibrosis, as well as increasing the probability of thrombosis due to platelet activation, and prompting coronary atherosclerosis [41]. However, the generation of TMAO from carnitine precursors does not fully explain their prognostic ability [40]. Nevertheless, in our study there were no significant differences in the occurrence of myocardial infraction and coronary artery disease between patients with resolved thrombus and those with persistent thrombus. Moreover, the group of patients characterized by lower levels of carnitine precursors had higher incidence of congestive heart failure.

Another factor contributing to disturbances in acylcarnitine level is antihypertensive treatment. Studies conducted on dogs in the last century have shown that the administration of metoprolol resulted in decreased activity of carnitine palmitoyltransferase I (CPT-I) and elevated triglyceride levels [42], potentially leading to lower ACs levels. Moreover, the use of beta-blockers is associated with impaired left atrial function (reservoir, conduit, and booster) in hypertensive patients, possibly due to their direct negative inotropic effects on the LA myocardium, dysfunction secondary to negative inotropic or lusitropic effects on the left ventricular (LV), and/or worsening of LA–LV–aortic coupling [43]. This impairment in LA function could potentially contribute to the risk of thrombus formation. Furthermore, another study showed that beta-blockers and ACE inhibitors/angiotensin II receptor antagonists do not appear to be protective against venous thromboembolism even though they were shown to reduce factor VIII:C (coagulation factor VIII) and enhance fibrinolysis, respectively [44]. However, antihypertensive treatment cannot explain the lower levels of ACs and the ineffective treatment with modified rivaroxaban dose in one of our study groups, as the percentage of individuals treated for hypertension was comparable in both responders and non-responders.

Additionally, the activity of the CPT-I enzyme, which is crucial for transporting fatty acids into mitochondria for β-oxidation, could also be reduced in chronic fatigue syndrome. A study comparing healthy controls (*n* = 49) with patients suffering from chronic fatigue syndrome (*n* = 44) revealed significantly altered levels of eight ACs (in most cases decreased) in the plasma of these patients, which could be the result of reduced CPT-I activity and consequently reduced mitochondrial β-oxidation. This reduction in CPT-I activity possibly results from the accumulation of omega-6 fatty acid observed in this patient population. There is currently no known cause of chronic fatigue syndrome, however, some studies suggest viral infections, which may alter fatty acid metabolism [45]. It is important to note that none of our patients underwent diagnosis for chronic fatigue syndrome. Even if our study groups included individuals suffering from chronic fatigue syndrome, based on the acylcarnitine profiles, it is highly unlikely that the distribution of these individuals differed between the two study groups.

**Figure A1:**
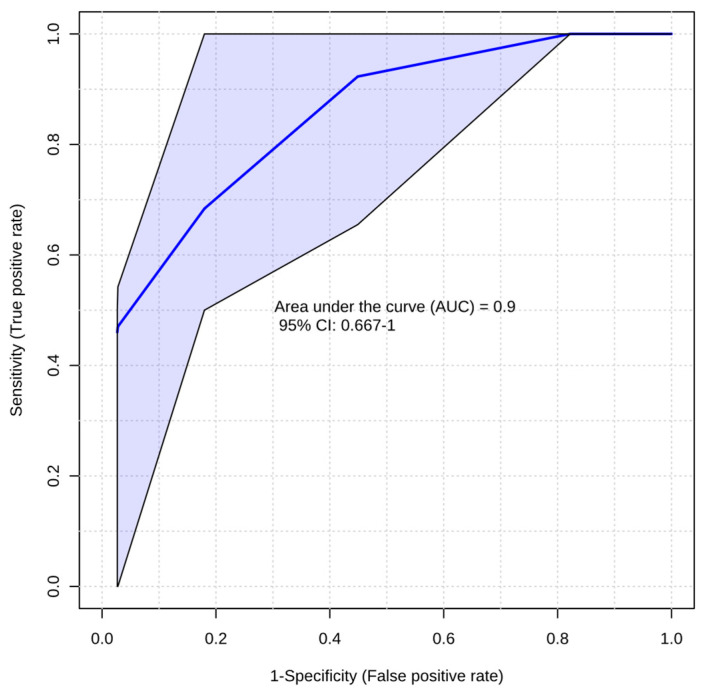
The area under receiver operating characteristic (ROC) curve for two variables selected based on PLS-DA algorithm was 0.90, with a specificity of 75% and a sensitivity of 86% for the prediction of thrombus resolution. The most frequently selected variables for the model were C6-DC (100%), iC5-OH (50%), and lysine (50%).

Despite the satisfactory area under the ROC curve (equal 0.90) analysis (Figure A1) with a specificity of 75% and a sensitivity of 86%, indicating strong predictive value of several analyzed ACs (mostly C6-DC, iC5-OH), carnitine, and its precursors (mostly lysine) for therapy effectiveness, prediction proved ineffective in some patients. It was observed that a subgroup of patients had persistent thrombosis despite having similar concentrations of carnitine, its precursors, and ACs (C3, C18, iC-5, iC5-OH, and C6-DC) in plasma, compared to a subgroup of patients in whom the thrombus was resolved. This indicates heterogeneity in factors influencing thrombus lysis and levels of carnitine, its precursors, and ACs, indicating that their levels may not be universally useful for prediction. As mentioned earlier, thrombus resolution is associated with higher LAA emptying flow velocities during AF, which in turn have been demonstrated to be influenced by ventricular heart rate, with longer cardiac cycles associated with higher mean LAA velocities. Other predictors of LAA thrombus resolution include young age and non-permanent AF [31]. A study conducted by Piotrowski et al. confirmed that the development of clots in the LAA is influenced by multiple factors, potentially encompassing the morphology of the LAA [12]. In addition, inflammation may also play a role. Several studies have indicated the potential role of systemic inflammation in promoting thrombus formation and potentially contributing to the onset of stroke and other types of thromboembolism in individuals with atrial fibrillation. The link between inflammation and thrombosis could be explained by increased CRP expression and alterations in platelet function observed during inflammation. Additionally, specific interactions between complement proteins and platelets, as well as the effects of proinflammatory cytokines, have been suggested. The association between inflammation and thrombosis could help to explain the clinical observation that while some patients experience thrombotic events despite receiving adequate anticoagulant therapy, others do not [46]. Another potential explanation for thrombus persistence despite anticoagulant treatment is that only fresh blood clots resolve under the influence of treatment, whereas organized clots do not respond, even to prolonged anticoagulation [47]. Moreover, studies have demonstrated that patients with a thrombus, who either received no prior anticoagulation treatment or received inadequate treatment (e.g., too short), show complete thrombus resolution efficacy of 100% when administered oral therapy (NOAC or VKA). However, if patients have previously received adequate anticoagulant treatment, the efficacy of oral medications in resolving thrombus is limited, with a success rate of only 43–44% [48].

Evaluating the groups of patients with resolved and persisted thrombus based on clinical and demographic characteristics, we observed a statistically significant difference in the incidence of IGT (*p* = 0.0384). The literature suggests the existence of a prothrombotic state with fibrinolytic dysfunction in individuals with IGT [49]. In our study, more cases of IGT were found in the group of patients with resolved thrombus. This may indicate that in some patients the standard 1 × 20 mg rivaroxaban dose is not effective in preventing thrombus formation, but the formed thrombus may be effectively resolved with the modified rivaroxaban treatment (2 × 15 mg). This modified dosage regimen, resulting in higher rivaroxaban blood concentrations, may effectively increase the anticoagulant effect, compensating for, e.g., fibrinolytic dysfunction associated with inflammation and/or lower levels of LCACs (C18), which exhibit anticoagulant properties. However, in other patients with thrombus, despite the standard 20 mg once-daily treatment, this increased anticoagulant effect due to modified rivaroxaban treatment (2 × 15 mg) may still prove ineffective, possibly due to reduced LAA emptying velocity, variations in thrombus morphology, and/or differences in prior anticoagulant treatment.

## 3. Materials and Methods

### 3.1. Study Group

The Local Ethics Committee of the Postgraduate Medical School, Warsaw, Poland (No. 49/PB/2015) approved the study protocol. Informed consent was obtained from all individual participants included in the study. All procedures performed in studies involving human participants were in accordance with the ethical standards of the institutional and/or national research committee and with the 1964 Helsinki declaration and its later amendments or comparable ethical standards. The inclusion criteria were age ≤ 80 years old, lack of advanced renal failure (creatinine clearance higher than 30 mL/min/1.73 m^2^ calculated using Cockcroft and Gault formula), no significant liver dysfunction (alanine transaminase level up to three times of the upper limit of the normal range) and no history of major bleeding, including intracranial hemorrhage. Women of childbearing potential were excluded unless an effective contraception was documented.

The study group consisted of 15 patients suffering from AF with thrombus in LAA in whom standard 20 mg once-daily treatment with rivaroxaban was ineffective in preventing the thrombus formation. Table 2 presents the clinical and demographic characteristics of these patients. They were selected from the group of 249 patients treated with rivaroxaban, as described previously in the RIVA-TWICE study [12]. Then, the dose was changed to 2 × 15 mg. Over the next 8 weeks the complete resolution of thrombus in the LAA was observed in 7 (46.7%) patients. EDTA-plasma samples were collected 3 h (corresponding to the average half-time of rivaroxaban), 12 h (time before the next dose in the 2 × 15 mg regimen) and 24 h (time before the next dose in the 1 × 20 mg regimen) before and after starting the 15 mg twice-daily dosing regimen. The rivaroxaban concentration was determined in all the plasma samples. For the metabolomic study only samples collected before the dosage regimen change (24 h) were used. Samples were stored at −80 until further analysis.

### 3.2. Sample Preparation

All chemicals were listed in Appendix A.1. Concentration of rivaroxaban in plasma was determined using QuEChERS method (details in Appendix A.2).

#### 3.2.1. Untargeted Metabolomics

Polar metabolites: A total of 150 µL of the internal standard solution (containing 1.3 µg/mL of each asymmetric dimethylarginine (ADMA-D6) and lysine-D9 in methanol–acetonitrile mixture (1:1, *v*/*v*)) was mixed with 50 µL of plasma. The samples were then incubated on ice for 10 min and centrifuged (10,000× *g*, 4 °C, 10 min) to remove precipitated proteins. Next, 100 µL aliquots of the resulting supernatants were transferred to the vials.

Nonpolar metabolites: A total of 150 µL of the internal standard solution (containing 0.65 µg/mL of each imipramine and indoxyl sulfate-D4 in isopropyl alcohol) was mixed with 50 µL of plasma. The samples were incubated on ice for 10 min and centrifuged (10,000× *g*, 4 °C, 10 min) to remove precipitated proteins. Next, 160 µL aliquots of the resulting supernatants were evaporated using a nitrogen blow drier (TurboVap, Biotage, Uppsala, Sweden). Extracts were resuspended in 160 µL of acetonitrile–water mixture (3:1, *v*/*v*) and centrifuged (10,000× *g*, 4 °C, 10 min). In total, 100 µL of supernatant was transferred into a vial and 30 µL of each extracted plasma sample was used to create a pooled quality control (QC) sample. Additionally, extract blank samples were prepared by substituting 50 µL of plasma with water. Both QC and blank samples were processed in the same way, as described above.

#### 3.2.2. Targeted Metabolomics

100 µL of plasma was mixed with 300 µL of acetonitrile. The samples were mixed thoroughly (1 min) and centrifuged (10,000× *g*, 4 °C, 5 min) to remove precipitated proteins. Next, 80 µL aliquots of the resulting supernatants were mixed with 20 µL of internal standards in acetonitrile (mixture of those bought as separate compounds at 5 µg/mL, and NSK-B-1 IS mix diluted 10-times) and analyzed.

### 3.3. Instrumental Methods

Rivaroxaban were determined using liquid chromatography coupled to mass spectrometry (LC-MS), separation was performed using an Agilent 1260 Infinity System (Agilent Technologies, Santa Clara, CA, USA), equipped with a degasser, an autosampler and a binary pump, coupled to a QTRAP 4000 hybrid triple quadrupole/linear ion trap mass spectrometer (AB Sciex, Framingham, MA, USA). Untargeted and targeted metabolomics were performed using a Dionex UltiMate 3000 UHPLC system connected to a Q-Exactive hybrid quadrupole-orbitrap mass spectrometer (Thermo Fisher Scientific, Waltham, MA, USA) equipped with heat electrospray ionization (HESI). The parameters of the methods were presented in Appendix A.3, Table A1.

### 3.4. Statistical Analysis

MetaboAnalyst 4.0 [50] was used for partial least squares-discriminant analysis (PLS-DA) and Quantitative Enrichment Analysis. The software was also employed to generate a heatmap using Euclidean distance as a measure of similarity and Ward’s method for clustering. Statistica 13 (licence: Medical University of Warsaw) was used to perform the ANOVA tests. Normal distribution and homogeneity of variance of the data were assessed using the Shapiro–Wilk test and Levene’s test, respectively. In the case of a lack of normal distribution or homogeneity of variances (*p* < 0.05), a subsequent statistical evaluation of significance was performed using the Mann–Whitney U (independent variables) or Wilcoxon matched-pairs test (dependent variables). Otherwise, Student’s *t*-test was used. In the case of qualitative variables, chi-square test was used to compare the groups. The predictive value of analyzed compounds was assessed by calculating the area under the receiver operating characteristic (ROC) curve.

## 4. Conclusions

The existing literature suggests that ACs concentrations can serve as a fingerprint across various disease entities. In this study, we have demonstrated for the first time that patients’ phenotype, specifically their carnitine, carnitine precursors, and ACs concentrations may serve as a predictive factor for the effectiveness of rivaroxaban treatment with a modified dose in the resolving of existing thrombus. Although a direct influence of ACs on thrombus resolution is highly unlikely, elevated levels of carnitine, its precursors, and ACs should be considered as an indicative feature of the patient’s phenotype. However, further research is needed. Studies focusing on the plasma concentrations of ACs in patients treated with the 1 × 20 mg dose of rivaroxaban, both effectively and non-effectively, would provide valuable insights into the potential clinical utility of this phenotype.

### Limitation

The limitation of our study is that patients in this group have multiple comorbidities, which may affect the results of the study. In addition, a detailed explanation of the mechanisms underlying the lack of response to treatment remains to be elucidated; this aspect has not been addressed in the present article.

## Figures and Tables

**Table 1:** Plasma concentrations (mean and standard deviation ^a^ or median and quartile range ^b^) of carnitine, its precursors and selected acylcarnitines in patients successfully and unsuccessfully treated with rivaroxaban at a dose of 15 mg/2× daily. Statistically significant differences are in bold.

Group	Compound	Concentration [ng/mL]		*p*-Value
		Patients with Persisting Thrombus (*n* = 8)	Patients in Whom Thrombus Was Resolved (*n* = 7)	
Carnitine precursors	Methionine ^a^	2304 (972)	3960 (1800)	***p* = ** **0.04601**
Carnitine precursors	Trimethyllysine ^b^	23 (25)	95 (56)	***p* = ** **0.01399**
Carnitine precursors	Butyrobetaine ^a^	117 (61)	277 (119)	***p* = ** **0.01090**
Carnitine precursors	Lysine ^b^	5940 (8460)	14,580 (23,400)	***p* = ** **0.04009**
Carnitine	CAR ^a^	3582 (1584)	5778 (1602)	***p* = ** **0.01958**
Short-chain ACs	C2 ^a^	1026 (360)	1350 (360)	NS, *p* = 0.10
Short-chain ACs	C3 ^a^	72 (31)	113 (34)	***p* = ** **0.02465**
Short-chain ACs	C4 ^a^	23.9 (8.1)	24.1 (8.1)	NS, *p* = 0.97
Medium-chain ACs	C8 ^b^	17 (13)	23 (29)	NS, *p* = 0.87
Medium-chain ACs	C12 ^a^	22 (11)	25 (18)	NS, *p* = 0.67
Long-chain ACs	C14 ^a^	8.6 (2.9)	8.8 (3.4)	NS, *p* = 0.85
Long-chain ACs	C16 ^a^	37 (10)	42.8 (9.5)	NS, *p* = 0.27
Long-chain ACs	C18 ^a^	10.3 (3.2)	14.4 (3.6)	***p* = ** **0.03833**
Branched-chain ACs	iC5 ^a^	20 (11)	30.8 (8.5)	***p* = ** **0.04578**
Unsaturated-chain ACs	C18:1 ^a^	180 (56)	185 (67)	NS, *p* = 0.85
Unsaturated-chain ACs	C18:2 ^a^	520 (173)	540 (252)	NS, *p* = 0.89
Hydroxyl ACs	C4-OH ^b^	56 (77)	146 (90)	NS, *p* = 0.09
Dicarboxyl ACs	C5-DC ^b^	9.9 (5.4)	34 (22)	NS, *p* = 0.12
Hydroxyl ACs	iC5-OH ^a^	117 (61)	277 (119)	***p* = ** **0.02278**
Dicarboxyl ACs	C6-DC ^b^	5940 (8460)	14,580 (23,400)	***p* = ** **0.00932**
Hydroxyl ACs	C16-OH ^a^	37 (10)	42.8 (9.5)	NS, *p* = 0.39

NS: not statistically significant (Student’s *t*-test ^a^ or Mann–Whitney U ^b^).

**Table 2:** Comparison of clinical and demographic characteristics between patients in whom thrombus persisted despite twice-daily rivaroxaban therapy and patients in whom thrombus was resolved. Statistically significant differences are in bold (*p* < 0.05).

Parameter	Patients with Persisting Thrombus (*n* = 8)	Patients in Whom Thrombus Was Resolved (*n* = 7)	*p*-Value
Age [years]	63.6 ± 9.7	62 ± 11	NS, *p* = 0.74
Man, *n* (%)	4 (50)	5 (71)	NS, *p* = 0.40
Height [m]	1.664 ± 0.088	1.70 ± 0.14	NS, *p* = 0.49
Weight [kg]	87 ± 17	87 ± 20	NS, *p* = 0.99
BMI [kg/m^2^]	31.2 ± 3.6	29.7 ± 3.4	NS, *p* = 0.40
ALT [U/I]	20.1 ± 5.8	19.6 ± 4.8	NS, *p* = 0.84
Creatinine [mg/dL]	1.00 ± 0.18	0.93 ± 0.11	NS, *p* = 0.37
eGFR C-G [mL/min]	85 ± 18	100 ± 37	NS, *p* = 0.32
Diabetes mellitus, *n* (%)	4 (50)	1 (14)	NS, *p* = 0.14
Impaired glucose tolerance, *n* (%)	0 (0)	3 (43)	***p* = ** **0.0384**
MI/CAD, *n* (%)	3 (38)	2 (29)	NS, *p* = 0.71
HA, *n* (%)	6 (75)	6 (86)	NS, *p* = 0.60
CHF, *n* (%)	6 (75)	1 (14)	***p* = ** **0.0187**
Smoking, *n* (%)	1 (13)	2 (29)	NS, *p* = 0.44
Beta blocker, *n* (%)	8 (100)	6 (86)	NS, *p* = 0.27
ACE-I, *n* (%)	8 (100)	6 (86)	NS, *p* = 0.27

ACE-I: angiotensin-converting-enzyme inhibitors; ALT: alanine aminotransferase; BMI: body mass index; CHF: congestive heart failure; eGFR C-G: estimated glomerular filtration rate by Cockcroft-Gault equation; MI/CAD: myocardial infarction/coronary artery disease, NS: not statistically significant (Student’s *t*-test or chi-square test (qualitative variables)). The data are presented as the mean ± standard deviation or as number and percentage (binary data).

## Data Availability

The raw data supporting the conclusions of this article will be made available by the authors on request.

## Abbreviations

The following abbreviations are used in this manuscript:

3Hyp	3-Hydroxyproline
4Hyp	4-Hydroxyproline
ACE	Angiotensin-converting enzyme
ACE-I	Angiotensin-converting-enzyme inhibitors
ACs	Acylcarnitines
ADMA-D6	Asymmetric dimethylarginine
AF	Atrial fibrillation
ALT	Alanine aminotransferase
AUC	Area under the curve
BBD	γ-Butyrobetaine dioxygenase
BMI	Body mass index
CAR	Carnitine
CHF	Congestive heart failure
C2	Acetylcarnitine
C3	Propionylcarnitine
C4	Butyrylcarnitine
C4-OH	3-Hydroksybutyrylcarnitine
C5-DC	Glutarylcarnitine
C6-DC	Methylglutarylcarnitine
C8	Octanoylcarnitine
C12	Dodecanoylcarnitine
C14	Myristoylcarnitine
C16	Palmitoylcarnitine
C16-OH	Hydroxypalmitoylcarnitine
C18	Octadecanoylcarnitine
C18:1	Oleoyl-L-carnitine
C18:2	Linoleoyl carnitine
CPT-I	Carnitine Palmitoyltransferase I
DG	Diglyceride
eGFR C-G	Estimated glomerular filtration rate by Cockcroft-Gault equation
ESC	European Society of Cardiology
FA	Fatty acid
FAO	Fatty acid oxidation
FDA	Food and Drug Administration
HESI	Heat electrospray ionization
IGT	Impaired Glucose Tolerance
IS	Internal standard
iC5	Isovalerylcarnitine
iC5-OH	Hydroxyisovalerylcarnitine
LA	Left atrial
LAA	Left atrial appendage
LCAC	Long-chain acylcarnitine
LC-MS	Liquid chromatography coupled to mass spectrometry
LLOQ	Lower limit of quantitation
MI/CAD	Myocardial infarction/coronary artery disease
NOAC	Non-vitamin K antagonist oral anticoagulant
NS	Not statistically significant
PE	Phosphatidylethanolamine
PLS-DA	Partial least squares-discriminant analysis
QC	Quality control sample
ROC	Receiver operating characteristic
SCAC	Short-chain acylcarnitine
TMAO	Trimethylamine N-oxide

## Appendix A

### Appendix A.1. Chemicals

Internal standards (ISs) for untargeted metabolomics: L-methionine-D4, indoxyl sulfate-D4, ADMA-D6, lysine-D9, targeted metabolomics: N-epsilontrimethyllysine-D9, gamma-butyrobetaine-D9 HCl, L-lysine-D9 HCl, and rivaroxaban-D4 were purchased from Toronto Research Chemicals (Toronto, ON, Canada). Other ISs for untargeted metabolomics included imipramine purchased from Sigma-Aldrich (St. Louis, MO, USA = Merck KGaA, Darmstadt, Germany), whereas for targeted metabolomics, NSK-B-1 (carnitine-D9 (free carnitine), acetylcarnitine-D3, propionylcarnitine-D3, butyrylcarnitine-D3, isovalerylcarnitine-D9, octanoylcarnitine-D3, myristoylcarnitine-D9, palmitoylcarnitine-D3) was bought from Cambridge Isotope Laboratories (Tewksbury, MA, USA). Analytical standards of rivaroxaban, oleoyl-L-carnitine (C18:1), linoleoyl carnitine (C18:2), 3-hydroksybutyrylcarnitine HCl (C4-OH), methylglutarylcarnitine HCl (C6-DC), gamma-butyrobetaine HCl, L-methionine, L-lysine, and N-epsilontrimethyllysine were from Toronto Research Chemicals (Toronto, ON, Canada). Standard mix NSKB US1 (Carnitine (free carnitine, CAR), acetylcarnitine (C2), propionylcarnitine (C3), butyrylcarnitine (C4), isovalerylcarnitine (iC5), octanoylcarnitine (C8), myristoylcarnitine (C14), palmitoylcarnitine (C16)), and NSK-B-G1-US1 (glutarylcarnitine (C5-DC), hydroxyisovalerylcarnitine (iC5-OH), dodecanoylcarnitine (C12), octadecanoylcarnitine (C18), and hydroxypalmitoylcarnitine (C16-OH)) were purchased from Cambridge Isotope Laboratories (Tewksbury, MA, USA). The solvents, methanol and acetonitrile hyper grade for LC-MS (LiChrosolv), isopropyl alcohol (IPA) for HPLC, and formic acid 98% were purchased from Merck (Darmstadt, Germany). Ultrapure water was obtained from a Millipore water purification system Milli-Q water (Burlington, MA, USA). The internal standard stock solutions were stored at −20 °C.

### Appendix A.2. Sample Preparation: Rivaroxaban Plasma Concentration

To determine the rivaroxaban concentration in plasma, the QuEChERS method was developed. In total, 100 μL of human plasma and 300 μL of the internal standard solution (Rivaroxaban-D4, 1.5 mg/L in acetonitrile) were added to the 1.5 mL polypropylene tube and mixed thoroughly. Then, 50 mg of ammonium acetate (or 100 and 200 mg in the optimization step) was added followed by vigorous shaking for 1 min. The entire mixture was put in the freezer (−20 °C) for 10 min and centrifuged for 1 min at 10,000× *g* at 4 °C. Then, 250 μL of the resulting supernatant was mixed with 25 mg of C18 sorbent for 1 min (or 50 mg C18 or 25 mg C18 + 25 mg PSA (primary secondary amine) in the optimization step), and centrifuged as previously described. The supernatant was diluted five times with water and transferred into the vial.

### Appendix A.3. Instrumental Methods

#### Appendix A.3.1. Rivaroxaban Concentration

The turbo ion spray source was operated in positive mode. The target compounds were analyzed in multiple reaction monitoring mode using the following transitions: *m*/*z* 436 > 145 (DP = 106 V, CE = 37 eV) for rivaroxaban and *m*/*z* 440 > 145 (DP = 111 V, CE = 37 eV) for rivaroxaban-D4. The chromatography was performed on an BionaCore C18 column (100 × 4.6 mm, 2.7 µm) (Merck, Darmstadt, Germany) maintained at 40 °C. The mobile phase consisted of solvent A (0.1% formic acid) and B (acetonitrile with 0.1% formic acid) delivered at 0.75 mL/min according to the following elution program (%B): 0 min 15%; 2 min, 15%, 6 min 90%, 13 min, 90%. The injection volume was 10 µL. The method was validated following the guideline of the European Medicines Agency in terms of: calibration range (1.95–1000 ng/mL), linearity (R^2^ >0.990), accuracy, and precision.

#### Appendix A.3.2. Untargeted Metabolomics

Chromatographic separation was conducted using two columns: SeQuant ZIC-HILIC column (100 mm × 2.1 mm, 5 µm) (Merck, Darmstadt, Germany) and Kinetex PFP column (100 mm × 4.6 mm, 2.6 µm) (Phenomenex, Torrance, CA, USA). The gradient elution in the first column was performed using mobile phase A = water, 0.1% formic acid, mobile phase B = acetonitrile, 0.1% formic acid, and gradient: t = 0.0, 90% B; t = 2.5, 90% B; t = 18.0, 50% B; t = 28.0, 50% B; t = 34.0, 90% B. All changes were linear and the flow rate was 0.50 mL/min. Column temperature was 40 °C and the injection volume was 5 µL. The gradient elution in the second column was performed using mobile phase A = water, 0.1% formic acid, mobile phase B = acetonitrile, 0.1% formic acid, and gradient: t = 0.0, 20% B; t = 2.0, 20% B; t = 20.0, 90% B; t = 30.0, 90% B; t = 31.0, 20% B; t = 35.0, 20% B. All changes were linear and the flow rate was 0.30 mL/min. Column temperature was 40 °C and the injection volume was 10 µL. QC samples were analyzed as the first ten injections and then every tenth injection with an additional five at the end of the analytical batch. Data were acquired in positive and negative ionization mode (70–1000 *m/z*) with a resolution of 70,000 (FWHM at *m/z* 200) using Thermo Xcalibur 3.0 software. Fragmentation was performed in different runs with a normalized collision energy of 20, 30, 40 eV. The ion selection threshold was 8 × 10^3^ counts, and the maximum allowed ion accumulation times were set to auto for both full MS scans and the tandem mass spectrum. Standard mass spectrometric conditions in all experiments were: spray voltage: 3.5 kV; sheath gas pressure: 60 arb; aux gas pressure: 20 arb; sweep gas pressure: 0 arb; heated capillary temperature: 320 °C; loop count: 3; isolation window: *m*/*z* 1.0; and dynamic exclusion: 6.0 s. For all full scan measurements, lock-mass ions from ambient air (*m*/*z* 445.1200 and 291.2842) were used as internal calibrants. The results obtained were analyzed using Compound Discoverer 3.0 software supplied by Thermo Fisher Scientific (Waltham, MA, USA).

#### Appendix A.3.3. Targeted Metabolomics

Targeted metabolomics was performed in positive mode. The target compounds were analyzed in multiple reaction monitoring modes using the transitions presented in Table A1. The chromatography was performed using a BionaCore C18 column (100 × 4.6 mm, 2.7 µm) and a SeQuant ZIC-HILIC column (50 mm × 2.1 mm, 5 µm) (Merck, Darmstadt, Germany) maintained at 40 °C. The mobile phase used for C18 column consisted of solvent A (water with 0.2% formic acid) and B (acetonitrile with 0.2% formic acid) delivered at 0.5 mL/min according to the following elution program (%B): 0 min 10%; 1 min, 10%, 6.5 min 90%, 12 min, 90%. Elution program for HILIC (%B): 0 min 90%; 1 min, 90%, 9 min 50%, 14 min, 50%, with mobile phase A consisting of a water solution of 20 mM ammonium acetate and mobile phase B consisting of acetonitrile with 0.2% formic acid. The injection volume was 10 µL in C18 and 5 µL in HILIC mode.

**Table A1:** The optimized parameters used in the MRM mode to determine compounds in ESI+.

Compound	*m*/*z*	DP [V]	CE [V]
Carnitine	162 > 103	66	23
Gamma-butyrobetaine	146 > 60	66	21
L-lysine	147 > 84	56	23
Trimethylysine	189 > 84	61	29
L-metionine	150 > 104	56	15
C2	204 > 85	71	27
C3	218 > 85	56	27
C4	232 > 85	71	29
C4-OH	248 > 85	56	31
C5-DC	276 > 85	61	35
iC5	246 > 85	71	29
iC5-OH	262 > 85	76	35
C6-DC	290 > 85	86	35
C8	288 > 85	91	33
C12	344 > 85	91	45
C14	372 > 85	101	47
C16	400 > 85	111	51
C16-OH	262 > 85	76	35
C18	428 > 85	131	73
C18:1	426 > 85	116	51
C18:2	424 > 85	86	49
Carnitine-D9	171 > 103	66	25
Gamma-butyrobetaine-D9	155 > 87	56	23
L-lysine-D9	156 > 93	66	25
Trimethylysine-D9	198 > 84	61	31
L-methionine-D4	154 > 137	56	13
Acetylcarnitine-D3	207 > 85	66	27
Butyrylcarnitine-D3	235 > 85	76	29
Isovalerylcarnitine-D9	255 > 85	76	31
Myristoylcarnitine-D9	381 > 85	121	55
Octanoylcarnitine-D3	291 > 85	91	33
Palmitoylcarnitine-D3	403 > 84	121	51
Propionylcarnitine-D3	221 > 85	66	29

DP: declustering potential; CE: collision energy.
